# Dose-dependent effects on rat liver miRNAs 200a/b and 429: potential early biomarkers of liver carcinogenesis

**DOI:** 10.1016/j.toxrep.2018.02.004

**Published:** 2018-02-19

**Authors:** S.M. Plummer, J. Wright, R.A. Currie

**Affiliations:** a MicroMatrices Associates Ltd, Dundee, UK; b Syngenta Ltd, Bracknell, UK

**Keywords:** Biomarker, microRNA, Liver, Hepatocarcinogesis

## Abstract

An increased incidence of liver tumours in the long term rodent bioassay is not an uncommon finding, invariably as a result of a non-genotoxic mode of action. Non-genotoxic liver carcinogenesis has been found to involve activation of certain nuclear hormone receptors (NHR) including the constitutive androstane receptor (CAR), peroxisome proliferator activated receptor alpha (PPARalpha) and arylhydrocarbon receptor (AHR) and more recently the induction of specific microRNAs (miRs), has also been demonstrated following CAR activation in studies up to 90 days (Koufaris et al., 2012). The stable induction of these tissue specific miRs, namely miR200a, 200b and 429, by liver non-genotoxic carcinogens may serve as early predictors (biomarkers) of heptocarcinogenic potential. To test this hypothesis we used RT-PCR to measure the levels of these miRs in the livers from Wistar rats treated with two rat hepatocarcinogenic and one non hepatocarcinogenic pyrazole carboxamide succinate dehydrogenase inhibitors, Isopyrazam, Sedaxane and Benzovindiflupyr, respectively. The miRs were quantified by RT-PCR in liver RNA samples from three 90 day repeat dose toxicity studies performed at the low, mid and high doses relative to control. In Isopyrazam treated rats a statistically significant (p < 0.01) dose-dependent increase in miR 200a, 220b and 429 in both males and females was observed, whilst for Sedaxane a significant (p < 0.05) increase in miR200b in males and females at the high dose was seen. Benzovindiflupyr treatment did not cause any dose related changes in miR 200a, 200b and 429 relative to control. Our results suggest that assessment of miR 200a/200b/429 levels has potential as a biomarker of the perturbation of pathways involved in hepatocarcinogenesis in Wistar rats. Further work is required to establish the possible relationship between miR200 cluster induction and CAR-mediated hepatocarcinogenesis in a more diverse range of compounds.

## 1 Introduction

The mechanisms of non-genotoxic carcinogenesis (NGC) involving activation of certain nuclear hormone receptors (NHR) including constitutive androstane receptor (CAR) peroxisome proliferator activated receptor alpha (PPARalpha) and arylhydrocarbon receptor (AHR) are relatively well characterised [[1], [2], [3], [4]]. However despite CAR activation being considered an initiating event in this process, [5], not all CAR activators are hepatocarcinogenic in long term rodent bioassays [6]. Hence CAR activation is considered necessary but not sufficient for hepatocarcinogenesis and certain CAR activators are more potent hepatocarcinogens than others. Biomarkers that could improve the prediction of the hepatocarcinogenic potential of CAR activator compounds would facilitate risk assessment.

MicroRNAs (miRs) in the miR 200a/200b/429 cluster are significantly induced in Fisher rat livers following 90 day dosing with phenobarbital (PB) [7], a prototypical CAR activator. It has also been demonstrated that miRs 200a and 200b were induced after 14 days dosing with PB, in a dose-dependent fashion and that this induction occurred only at PB doses that are carcinogenic but not at non-carcinogenic doses [8]. A recent study by Romer and co-workers also identified a miR ‘signature’ including miR 200a/200b/429 cluster members distinguishing between hepatocarcinogenic and non-hepatocarcinogenic compounds in Wistar rats dosed for 14 days, suggesting that these miRs could serve as predictors of hepatocarcinogenic potential [9].

To further test this hypothesis we have measured miRs 200a/200b/429 following 90 days treatment over a range of doses with a class of pyrazole carboxamide succinate dehydrogenase inhibitor (SDHI) compounds including two rat hepatocarcinogens (Isopyrazam and Sedaxane) and one SDHI that was non-hepatocarcinogenic in rats (Benzovindiflupyr) [[10], [11], [12]].

## 2 Materials and methods

### 2.1 Samples

The samples used in this study were liver formalin fixed paraffin embedded (FFPE) blocks derived from 3 previous rat 90 day repeat dose (3 doses) dietary toxicity studies [[13], [14], [15]] with 3 different test compounds: Isopyrazam (3-(difluoromethyl)-1-methyl-*N*-1,2,3,4-tetrahydro-9-isopropyl-1,4-methanonaphthalen-5-yl]pyrazole-4-carboxamide), Sedaxane (*N*-[2-[1,1′-bicyclopropyl]-2-ylphenyl]-3-(difluoromethyl)-1-methyl-1H-pyrazole-4-carboxamide) and Benzovindiflupyr(*N*-9-(dichloromethylene)-1,2,3,4-tetrahydro-1,4-methanonaphthalen-5-yl]-3-(difluoromethyl) 1-methylpyrazole-4-carboxamide).

5 males and 5 females from each group, control, low, medium and high dose were analysed for miR 220a/200b/429 levels from each study. Four 20 μM sections were cut from each of the liver FFPE blocks and sections of the left lateral lobe were placed in a 1.5 ml microtube prior to RNA extraction.

### 2.2 RNA extraction and QC

RNA extraction was performed according to the ABI RecoverAll™ Total Nucleic Acid Isolation Kit protocol (Part Number 1975 M Rev. C 02/2011). RNA QC 260/280 nm and 230/260 nm absorbance (ΔA) ratios were measured using a NanoDrop™ spectrophotometer.

### 2.3 RT-PCR analysis

100 ng total liver RNA extracted using the Recoverall FFPE extraction kit (Ambion cat no AM1975) was converted from miRNA into cDNA in singleplex reactions using the Taqman reverse transcription kit (ABI cat no 4366596) with the 4 specific stem loop primers [001718 (snoRNA), 00502 (miRBase ID: rno-miR-200a-3p), 002274 (miRBase ID: rno-miR-200b-5p), 001077 (miRBAse ID: rno-miR-429)] that amplify mature miRNAs but not precursors. PCR reaction efficiency was tested for each of the 4 targets (snoRNA endogenous control and 3 miRs) by constructing standard curves. Standard curves were made for each of the targets using a pooled control RNA/cDNA sample made from a pool of RNA samples of control males and females from all 3 studies; and 9 separate pools (males and females) from each of the low, intermediate and high dose treatment groups from the 3 studies. Each of the 10 cDNAs were added to qPCR reactions in a range of concentrations (100 ng down to 0.01 ng) for each of the 4 targets. The slopes of the curves were checked to assess whether or not they fell between −3.10 and −3.60, to evaluate whether the PCR amplicons doubled every cycle. The slope was used to assess the amplification efficiency. This process also identified how much input cDNA would give efficient amplification across all samples and probes. The cycle threshold (Ct) was checked to determine whether or not it varied by more than 0.5 between untreated and treated samples. This was performed to check that the representation of the endogenous control RNA(s) was consistent across all the samples. Singleplex Taqman microRNA assay reactions for miRs 200a, 200b and 429 were performed on 10 ng of each of the cDNA samples in duplicate using the probe specific Taqman primers.

### 2.4 Data analysis and statistics

The data were processed using a method which corrected for variations in PCR efficiencies [16]. Fold change values (R values) were calculated for the controls as well as the treated samples relative to a pooled control (calibrator) using the Pfaffl equation:

Ratio(R) = (Etarget) ^ΔCttarget(calibrator−sample)^/(Ereference) ^ΔCtreference(calibrator−sample)^, [16].

Where: Etarget = PCR efficiency of the target (i.e. miR 200a, 200b or 429); Ereference = PCR efficiency of the reference gene i.e snoRNA; E = 10^[−1/slope]^; Slope = slope of the standard curve plots of Ct (x axis) vs Log inital RNA/cDNA quantity, and ΔCt target (calibrator − sample) = (Ct target) in the calibrator (pooled control) − (Ct target) in the sample (control or treated). **NB** for 100% efficiency the slope is −3.32 because a 10 fold amplification will take 3.32 PCR cycles (2^3.32^ = 10). Hence slope = −3.32/1 (Log 10 = 1) = −3.32. The efficiency values were calculated from the slopes of standard curve plots of Ct vs Log input cDNA (0.01 ng–100 ng).

A ‘calibrator’ Ct value was determined for each of the miR targets including snoRNA by using cDNA derived from reverse transcription of a pooled control RNA sample made up of equal amounts of RNA from male and female control samples from all the treatment groups. Ct values derived from this sample were used for normalising R values using the Pfaffl equation (see above).

The calibrator Ct value was employed so that data could be normalised across different TaqMan plates. This method facilitated determination of R (fold change) values for controls as well as treated samples. As there were differences in the control (basal) levels of the miRs between males and females the data (fold change values) were also expressed relative to their sex matched controls for the purposes of comparing males vs females. This was performed by dividing the control and treated R values with their respective sex matched control R value as follows: male treated R/male control R or female treated R/female control R.

Statistical comparisons of the R value data were performed for treated samples against their respective controls. Statistical comparisons of individual group data (R values) vs respective (sex matched) controls were performed with a two tailed Student’s T test using MS Excel. Statistical comparisons across all treatment groups were performed with one way ANOVA with post hoc Scheffe’s test using Stat Plus software.

## 3 Results

### 3.1 Effects of SDHI treatment on miR levels

**Fig. 1:**
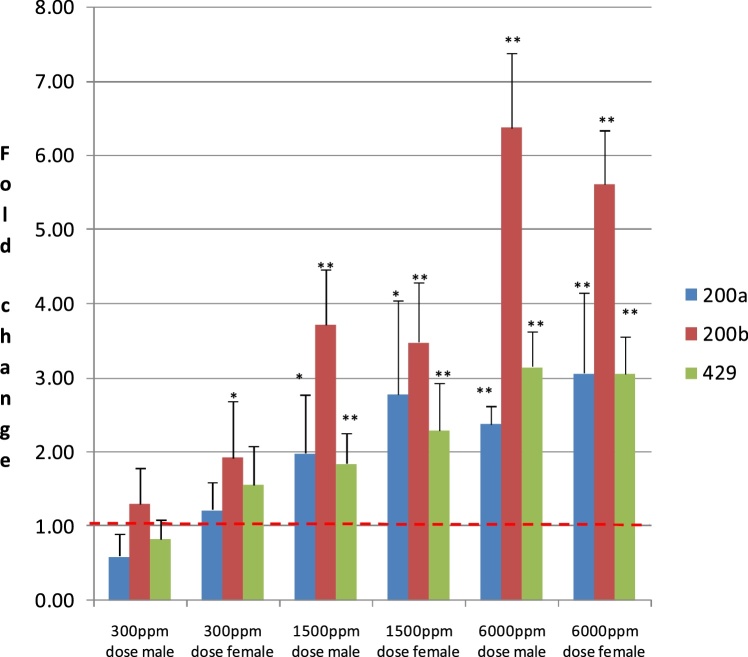
Relative Expression of MiR 200a, 200b, 429 in Liver of Rats Treated for 90 days with Isopyrazam. Relative expression of miR 200a, 200b and 429 in liver (left lobe) of rats treated (dietary) for 90 days with Isopyrazam (low dose: 300 ppm; mid dose: 1500 ppm; high dose: 6000 ppm). Results are mean (n = 5) ± SD fold changes relative to sex matched controls. *, ** significantly different from control by Student’s T test, p < 0.05 or p < 0.01, respectively. **NB**: Data are normalised to the respective sex matched controls in order to aid visual comparison of the data. Dashed red line shows control value = 1.

With Isopyrazam there was a dose-dependent increase in miR 200a, 220b and 429 in males and females (Fig. 1, supplementary data Table 1). All 3 miRs were significantly higher vs control (p < 0.01 by students T test) at low, mid and high dose for females and were significantly higher vs control (p < 0.05) at mid and high dose for males.

**Fig. 2:**
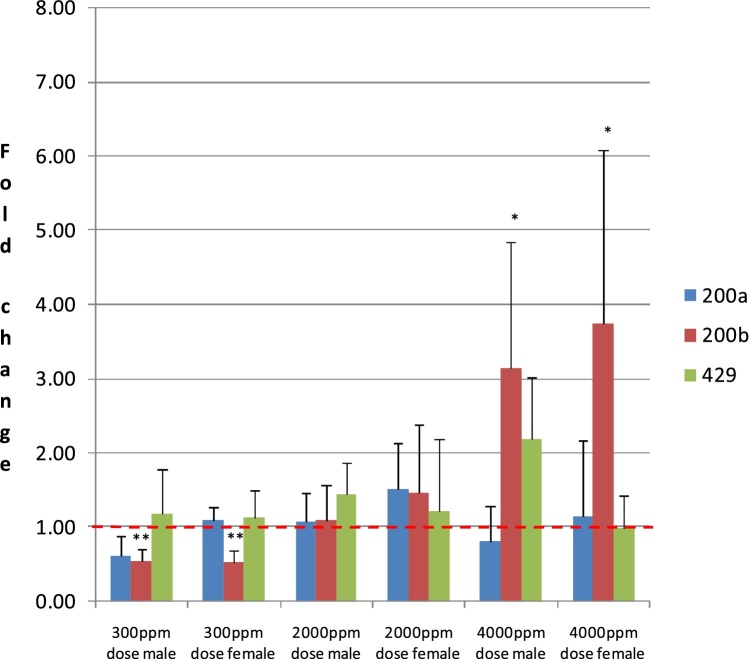
Relative Expression of MiR 200a, 200b, 429 in Liver of Rats Treated for 90 days with Sedaxane. Relative expression of miR 200a, 200b and 429 in liver (left lobe) of rats treated (dietary) for 90 days with Sedaxane (low dose: 300 ppm; mid dose: 2000 ppm; high dose: 4000 ppm). Results are mean (n = 5) ± SD fold changes relative to sex matched controls. *, ** significantly different from control by Student’s T test, p < 0.05 or p < 0.01, respectively. **NB**: Data are normalised to the respective sex matched controls in order to aid visual comparison of the data. Dashed red line shows control value = 1.

With Sedaxane treatment miR 200b was significantly (p < 0.05) higher vs control at the high dose in males and females (Fig. 2, supplementary data Table 1). At the low dose of Sedaxane miR 200b was significantly (p < 0.01) lower vs control in males and females (Figs. 2, supplementary data Table 1). There was no change in either miR200a or miR 429 following Sedaxane treatment, (Fig. 2).

**Fig. 3:**
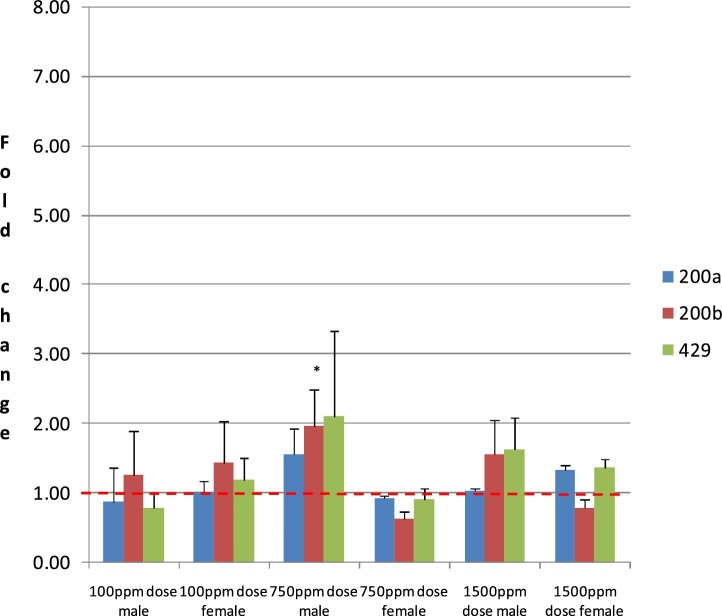
Relative Expression of MiR 200a, 200b, 429 in Liver of Rats Treated for 90 days with Benzovindiflupyr. Relative expression of miR 200a, 200b and 429 in liver (left lobe) of rats treated (dietary) for 90 days with Benzovindiflupyr (low dose: 100 ppm; med dose: 750 ppm; high dose; 1500 ppm). Results are mean (n = 5) ± SD fold changes relative to sex matched controls. *significantly different from control by Student’s T test, p < 0.05. **NB**: Data are normalised to the respective sex matched controls in order to aid visual comparison of the data. Dashed red line shows control value = 1.

With Benzovindiflupyr treatment there were no significant treatment related changes in miR 200a, 200b or 429. There was a slight increase in miR 200b vs control at the mid dose in males, which achieved statistical significance (p < 0.05), however due to the lack of a dose-response and the lack of an effect in females this change was not considered treatment related (Figs. 3, supplementary data Table 1).

### 3.2 PCR amplification efficiencies and intra- and inter-assay variation

PCR amplification efficiencies for the target transcripts snoRNA, miR 200a, miR 200b and miR 429 varied between 78%-100% efficiency (Suplementary data Table 3) and the inter-assay variation (%CV) was <1.5% and intra-assay variation was <2.5% for all targets (Supplementary data Table 4)

### 3.3 Incidence of hepatocellular tumours in wistar rats treated with the SDHIs

We evaluated the miR 200a/b and 429 induction data for the three SDHI compounds in the context of previously generated hepatocarcinogenicity data derived from 104 week feeding studies in Wistar rats [[13], [17], [18]]. Isopyrazam resulted in a significantly higher incidence of hepatocellular adenomas in female Wistar rats at the high dose (3000 ppm), Table 1. Sedaxane was deemed to be a weak hepatocarcinogen because it caused a slight (not statistically significant) increase in adenomas at the high dose. By contrast Benzovindiflupyr treatment did not cause a significant increase in hepatocellular tumours in Wistar rats (either sex) (Table 1). As miR 200b was significantly induced by Isopyrazam and Sedaxane in males and females there is concordance between the effects on this miR alone and the incidence of rat hepatocellular tumours. However as Isopyrazam, unlike Sedaxane and Benzovindiflupyr, also caused a dose-dependent induction of miR200a and miR429, the effects of the SDHIs on all three miR 200a/22b/429 cluster members is concordant with its increased potency relative to Sedaxane at the level of hepatocellular tumours.

**Table 1:** Incidence of hepatocellular tumours at terminal kill and preterm in male and female Wistar rats fed Isopyrazam, Sedaxane or Benzovindiflupyr in diet for 104 weeks.

	Incidence^a^							
	Males (n = 52)				Females (n = 52)			
**Isopyrazam**								
Dietary concentration (ppm)	0 ppm	100 ppm	500 ppm	3000 ppm	0 ppm	100 ppm	500 ppm	3000 ppm
Number of animals examined	52	52	52	52	52	52	52	52
Hepatocellular hypertrophy	0	12**	45**	49**	0	22**	49**	50**
Hepatocellular adenoma	1	0	0	3	0	1	1	11**
Hepatocellular carcinoma	0	0	0	1	0	0	0	1

**Sedaxane**								
Dietary concentration (ppm)	0 ppm	200 ppm	1200 ppm	3600 ppm	0 ppm	200 ppm	1200 ppm	3600 ppm
Number of animals examined	52	52	52	52	52	52	52	52
Hepatocellular hypertrophy	0	0	8**	27***	0	0	1	50***
Hepatocellular adenoma	1	1	1	5	0	2	2	2
Hepatocellular carcinoma	0	0	0	0	0	0	0	0

**Benzovindiflupyr**								
Dietary concentration (ppm)	0 ppm	25 ppm	100 ppm	600 ppm	0 ppm	25 ppm	100 ppm	400 ppm
Number of animals examined	52	52	52	52	52	52	52	52
Heptocellular Hypertrophy	0	1	8**	13**	0	2	5	36**
Hepatocellular adenoma	1	0	0	1	1	1	1	3
Hepatocellular carcinoma	0	0	0	0	0	0	0	0

** p < 0.01.

*** P < 0.001 (Mann-Whitney test).

a Sources: JMPR [[10], [11], [12]], Milburn [17], Perry [18], MacKay [13].

Microscopic examination of the livers from the carcinogenicity studies also revealed a dose related centrilobular hepatocyte hypertrophy in both males and females for all three compounds, most pronounced with Isopyrazam (Table 1).

In the 90 day studies similar centrilobular hepatocellular hypertrophy, typical of CAR activators, was observed for all three compounds [[10], [11], [12]].

## 4 Discussion

In the present study we tested a hypothesis that the induction of miR 200a, 200b and 429 expression may distinguish between succinate dehydrogenase inhibitor (SDHI) compounds (Isopyrazam and Sedaxane) known to produce rat liver tumours and one structurally related SDHI compound which did not (Benzovindiflupyr). Only Isopyrazam, the more potent hepatocarcinogenic compound in this group of compounds, demonstrated significant changes to all three miR 200a/200b/429 cluster members, suggesting the potential for these miRs as indicators of hepatocarcinogenic potency.

As only Isopyrazam and Sedaxane but not Benzovindiflupyr caused rat liver tumours there is concordance between the effect of the SDHIs on the three miRs and their effects at the level of hepatocellular carcinogenesis. Since the doses of Isopyrazam in the 90 day study (300 ppm, 1500 ppm and 6000 ppm) were higher than those in the 104 week study (100 ppm, 500 ppm and 3000 ppm) it is not possible to assess whether or not there is dose concordance between the effects of Isopyrazam on the miRs and its effect at the level of hepatocellular tumours. However as the miRs were induced by Isopyrazam at all three doses in females but only at the mid and high doses in males the data indicates that the effect on the miRs is more pronounced in females than in males which concurs with an increased potency of this compound at the level of hepatocellular tumours in females.

The induction of miRs 200a/200b/429 cluster members by the prototypical CAR activator PB correlates with the liver switching from a proliferative to a non-proliferative state [8]. In tumours the transcriptional repressor ZEB1 has been found to repress miRs 200a 200b and 429 [[19], [20]] that are thought to function to maintain epithelial differentiation, and ZEB1 and miR200 are interconnected via a double negative feedback loop [21]. Hence it has been suggested that induction of these miRs by PB could form part of a response to maintain the differentiated state of hepatocytes under a proliferative stimulus [7]. As this suggests that the induction of these miRs forms part of an adaptive/protective response to a procarcinogenic stimulus, it is possible that the ability to initiate this response could modulate carcinogenic susceptibility and could potentially mediate species differences in the hepatocarcinogenic effects of CAR activator compounds. At present there is no published data indicating whether or not CAR activator compounds will induce miR200a/200b/429 cluster members in mice. The induction response of miR 200b has previously been found to be a determinant of strain-specific susceptibility to the development of non alcoholic fatty liver disease (NASH) in C57BL/6J and DBA/2J mice [22].

As the study was confined to a limited set of CAR activator compounds that were structurally unrelated to the compounds tested by Koufaris and coworkers [7], it will be of interest to establish whether or not our findings are consistent in other structurally unrelated compounds that fall into the ‘CAR activator’ category and also to explore the possibility that induction of these miRs might be predictive of hepatocarcinogenesis in compounds that act via other NHRs such as peroxisome proliferator receptor alpha (PPARalpha) an aryl hydrocarbon receptor (AHR) beyond those described in previous studies [[7], [9]].
