# Supplementary material for: Dose-dependent effects on rat liver miRNAs 200a/b and 429: potential early biomarkers of liver carcinogenesis
Source: Toxicol Rep. 2018 Feb 19;5:309–13. doi: 10.1016/j.toxrep.2018.02.004 (PMC5856664; doi:10.1016/j.toxrep.2018.02.004)
Supplement: Supplementary file 1 [file mmc1.docx]

**Supplementary data:**

Supplementary data Table 1. Effects of the treatments Isopyrazam, Sedaxane and Benzovindiflupyr expressed in terms of fold change relative to pooled control (R values) for each of the three Rn miRs (200a, 200b and 429) as calculated using the Pfaffl equation.

| **Rn miR 200a** | **Sample size** | **Mean (R value)** | **SD (R value)** | **Fold change normalised to sex matched control** | **SD (Fold change**  **normalised to sex**  **matched control)** |
| --- | --- | --- | --- | --- | --- |
| Isopyrazam control male | 5 | 0.67 | 0.41 | 1 | 0.61 |
| Isopyrazamcontrol female | 5 | 0.30 | 0.07 | 1 | 0.22 |
| Isopyrazam Low dose male | 5 | 0.40 | 0.21 | 0.60 | 0.31 |
| IsopyrazamLow dose female | 5 | 0.36 | 0.11 | 1.23 | 0.37 |
| Isopyrazam Med dose male | 5 | 1.33 * | 0.52 | 1.99 | 0.78 |
| Isopyrazam Med dose female | 5 | 0.83* | 0.38 | 2.78 | 1.27 |
| IsopyrazamHigh dose male | 5 | 1.59** | 0.16 | 2.38 | 0.25 |
| Isopyrazam High dose female | 5 | 0.91** | 0.32 | 3.07 | 1.08 |
| Sedaxane control male | 5 | 1.67 | 0.48 | 1 | 0.29 |
| Sedaxane control female | 4 | 0.72 | 0.16 | 1 | 0.22 |
| Sedaxane low dose male | 5 | 1.02 | 0.44 | 0.61 | 0.26 |
| Sedaxane low dose female | 5 | 0.80 | 0.12 | 1.10 | 0.17 |
| Sedaxane med dose male | 5 | 1.81 | 0.63 | 1.08 | 0.38 |
| Sedaxane med dose female | 5 | 1.09 | 0.45 | 1.52 | 0.62 |
| Sedaxane high dose male | 5 | 1.35 | 0.81 | 0.81 | 0.49 |
| Sedaxane high dose female | 5 | 0.83 | 0.75 | 1.15 | 1.03 |
| Benzovindiflupyr control male | 5 | 1.16 | 0.50 | 1 | 0.43 |
| Benzovindiflupyr control female | 5 | 1.16 | 0.52 | 1 | 0.45 |
| Benzovindiflupyr low dose male | 5 | 1.02 | 0.58 | 0.88 | 0.50 |
| Benzovindiflupyr low dose female | 5 | 1.18 | 0.19 | 1.02 | 0.16 |
| Benzovindiflupyr med dose male | 5 | 1.81 | 0.43 | 1.56 | 0.37 |
| Benzovindiflupyr med dose female | 5 | 1.07 | 0.04 | 0.92 | 0.04 |
| Benzovindiflupyr high dose male | 4 | 1.59 | 0.05 | 1.04 | 0.04 |
| Benzovindiflupyr high dose female | 5 | 1.21 | 0.07 | 1.44 | 0.08 |
| **Rn miR 200b** |  |  |  |  |  |
| Isopyrazam control male | 5 | 0.88 | 0.40 | 1 | 0.41 |
| Isopyrazamcontrol female | 5 | 0.40 | 0.15 | 1 | 0.36 |
| Isopyrazam Low dose male | 5 | 1.15 | 0.43 | 1.30 | 0.49 |
| IsopyrazamLow dose female | 5 | 0.77* | 0.30 | 1.93 | 0.77 |
| Isopyrazam Med dose male | 5 | 3.27** | 0.65 | 3.72 | 0.74 |
| Isopyrazam Med dose female | 5 | 1.38** | 0.32 | 3.49 | 0.81 |
| IsopyrazamHigh dose male | 5 | 5.62** | 0.89 | 6.38 | 1.01 |
| Isopyrazam High dose female | 5 | 2.22** | 0.29 | 5.61 | 0.73 |
| Sedaxane control male | 5 | 1.64 | 0.61 | 1 | 0.37 |
| Sedaxane control female | 5 | 0.61 | 0.14 | 1 | 0.22 |
| Sedaxane low dose male | 5 | 0.89* | 0.26 | 0.54 | 0.16 |
| Sedaxane low dose female | 5 | 0.33** | 0.09 | 0.54 | 0.15 |
| Sedaxane med dose male | 5 | 1.81 | 0.76 | 1.10 | 0.47 |
| Sedaxane med dose female | 5 | 0.89 | 0.57 | 1.46 | 0.93 |
| Sedaxane high dose male | 5 | 5.15* | 2.81 | 3.14 | 1.71 |
| Sedaxane high dose female | 5 | 2.29* | 1.42 | 3.75 | 2.33 |
| Benzovindiflupyr control male | 4 | 1.21 | 0.80 | 1 | 0.66 |
| Benzovindiflupyr control female | 5 | 1.16 | 0.57 | 1 | 0.49 |
| Benzovindiflupyr low dose male | 5 | 1.53 | 0.78 | 1.26 | 0.64 |
| Benzovindiflupyr low dose female | 5 | 1.68 | 0.69 | 1.44 | 0.59 |
| Benzovindiflupyr med dose male | 5 | 2.38* | 0.65 | 1.97 | 0.53 |
| Benzovindiflupyr med dose female | 5 | 0.74 | 0.11 | 0.64 | 0.09 |
| Benzovindiflupyr high dose male | 5 | 1.89 | 0.60 | 1.56 | 0.49 |
| Benzovindiflupyr high dose female | 5 | 0.92 | 0.14 | 0.79 | 0.12 |
| **Rn miR429** |  |  |  |  |  |
| Isopyrazam control male | 5 | 1.23 | 0.50 | 1 | 0.41 |
| Isopyrazamcontrol female | 5 | 0.56 | 0.13 | 1 | 0.24 |
| Isopyrazam Low dose male | 5 | 1.02 | 0.32 | 0.83 | 0.26 |
| IsopyrazamLow dose female | 5 | 0.88 | 0.29 | 1.56 | 0.52 |
| Isopyrazam Med dose male | 5 | 2.27** | 0.51 | 1.85 | 0.41 |
| Isopyrazam Med dose female | 5 | 1.29** | 0.36 | 2.29 | 0.65 |
| IsopyrazamHigh dose male | 5 | 3.88** | 0.58 | 3.16 | 0.47 |
| Isopyrazam High dose female | 5 | 1.72** | 0.29 | 3.05 | 0.51 |
| Sedaxane control male | 5 | 1.23 | 0.50 | 1 | 0.41 |
| Sedaxane control female | 5 | 1.37 | 0.70 | 1 | 0.51 |
| Sedaxane low dose male | 5 | 0.97 | 0.18 | 1 | 0.19 |
| Sedaxane low dose female | 5 | 1.64 | 0.82 | 1.19 | 0.60 |
| Sedaxane med dose male | 5 | 1.08 | 0.37 | 1.12 | 0.38 |
| Sedaxane med dose female | 5 | 1.98 | 0.59 | 1.44 | 0.43 |
| Sedaxane high dose male | 5 | 1.17 | 0.94 | 1.21 | 0.97 |
| Sedaxane high dose female | 5 | 3.01* | 1.14 | 2.20 | 0.83 |
| Benzovindiflupyr control male | 5 | 0.96 | 0.42 | 0.99 | 0.43 |
| Benzovindiflupyr control female | 5 | 1.67 | 0.55 | 1 | 0.33 |
| Benzovindiflupyr low dose male | 5 | 1.67 | 0.48 | 1 | 0.28 |
| Benzovindiflupyr low dose female | 4 | 1.31 | 0.36 | 0.78 | 0.22 |
| Benzovindiflupyr med dose male | 5 | 1.99 | 0.53 | 1.19 | 0.31 |
| Benzovindiflupyr med dose female | 5 | 3.51 | 2.06 | 2.10 | 1.23 |
| Benzovindiflupyr high dose male | 5 | 1.53 | 0.27 | 0.91 | 0.16 |
| Benzovindiflupyr high dose female | 4 | 2.72 | 0.76 | 1.63 | 0.46 |
| Isopyrazam control male | 5 | 2.30 | 0.19 | 1.38 | 0.11 |

R values and normalised fold change values are means ± SD (n=5). R values are ratio values calculated using the Pfaffl equation. The normalised fold change values (expressed relative to sex match controls) were used to facilitate a comparison between males and females. *,** significantly different from respective sex matched controls by student’s T test , p≤0.05 or P<0.01, respectively.

Supplementary data Table 2: Details of Animals numbers/samples used for miRNA RT-PCR analysis

| **Group** | **Treatment** | **Number of rats per group** | | **Liver left lobe FFPE sections/RNA sample** |
| --- | --- | --- | --- | --- |
|  |  | **Male** | **Female** |  |
|  | **Isopyrazam** |  |  |  |
| 1 | Control | 5 | 5 | 4 |
| 2 | Low dose (300ppm) | 5 | 5 | 4 |
| 3 | Med dose (1500ppm) | 5 | 5 | 4 |
| 4 | High dose (6000ppm) | 5 | 5 | 4 |
|  |  |  |  |  |
|  | **Sedaxane** |  |  |  |
| 1 | Control | 5 | 5 | 4 |
| 2 | Low dose (300 ppm) | 5 | 5 | 4 |
| 3 | Med dose (2000 ppm) | 5 | 5 | 4 |
| 4 | High dose (4000 ppm) | 5 | 5 | 4 |
|  |  |  |  |  |
|  | **Benzovindiflupyr** |  |  |  |
| 1 | Control | 5 | 5 | 4 |
| 2 | Low dose (150 ppm) | 5 | 5 | 4 |
| 3 | Med dose  (750 ppm) | 5 | 5 | 4 |
| 4 | High dose (1500 ppm) | 5 | 5 | 4 |

## Suplementary data Table 3: Amplification efficiencies of snoRNA and miR 200a, 200b and 429 RT-PCR reactions .

Amplification efficiencies were calculated as a % using the following equation: Efficiency (%) E%=10^(-1/slope)^-1x100 (%). **Note**: Calculation of R values with the Pfaffl equation (Pfaffl 2001) used E values calculated from the slope of Ct value vs cDNA input curves as follows: E=10^(-1/slope)^ .

|  | **snoRNA** | | | **Rn miR 200a** | | | **Rn miR 200b** | | | **Rn miR 429** | | |
| --- | --- | --- | --- | --- | --- | --- | --- | --- | --- | --- | --- | --- |
| **Standard Curve** | **Slope** | **Amplification Efficiency (%) E%=10(-1/slope)-1x100 (%)** | **Efficiency (E=10^(-1/slope)^) (Pfaffl et al. 2001)** | **Slope** | **Amplification Efficiency (%) E%=10(-1/slope)-1x100 (%)** | **Efficiency (E=10(-1/slope)) (Pfaffl et al. 2001)** | **Slope** | **Amplification Efficiency (%) E%=10(-1/slope)-1x100 (%)** | **Efficiency (E=10(-1/slope)) (Pfaffl et al. 2001)** | **Slope** | **Amplification Efficiency (%) E%=10(-1/slope)-1x100 (%)** | **Efficiency (E=10(-1/slope)) (Pfaffl et al. 2001)** |
| **1 Pooled Controls** | -3.68 | 86.96 | 1.87 | -3.47 | 94.17 | 1.94 | -3.00 | 100.92 | 2.15 | -3.60 | 89.57 | 1.90 |
| **Isopyrazam male & female low dose** | -3.97 | 78.60 | 1.79 | -3.20 | 105.35 | 2.05 | -3.59 | 89.91 | 1.90 | -3.30 | 100.92 | 2.01 |
| **Isopyrazam male & female med dose** | -3.92 | 79.93 | 1.80 | -3.40 | 96.84 | 1.97 | -4.00 | 77.82 | 1.78 | -3.57 | 90.59 | 1.91 |
| **Isopyrazam male & female high dose** | -3.50 | 93.07 | 1.93 | -3.40 | 96.84 | 1.97 | -3.68 | 86.95 | 1.87 | -3.39 | 89.57 | 1.97 |
| **Sedaxane male& female low dose** | -3.60 | 89.57 | 1.90 | -3.47 | 94.17 | 1.94 | -2.77 | 129.62 | 2.30 | -3.30 | 100.92 | 2.01 |
| **Sedaxane male & female med dose** | -3.97 | 78.60 | 1.79 | -3.78 | 83.88 | 1.84 | -3.53 | 91.99 | 1.92 | -3.32 | 100.08 | 2.00 |
| **Sedaxane male & female high dose** | -3.27 | 102.21 | 2.02 | -3.93 | 79.66 | 1.80 | -3.22 | 104.44 | 2.04 | -3.62 | 88.90 | 1.89 |
| **Benzovindiflupyr male & female low dose** | -3.39 | 97.24 | 1.97 | -3.82 | 82.71 | 1.83 | -3.22 | 104.44 | 2.04 | -3.70 | 86.32 | 1.86 |
| **Benzovindiflupyr male & female med dose** | -3.90 | 80.47 | 1.80 | -3.80 | 83.29 | 1.83 | -3.96 | 78.86 | 1.79 | -3.52 | 96.84 | 1.92 |
| **Benzovindiflupyr male & female high dose** | -3.94 | 79.39 | 1.79 | -3.39 | 97.23 | 1.97 | -3.53 | 91.99 | 1.92 | -3.49 | 93.43 | 1.93 |

## Supplementary data Table 4 A-D: Intra and Inter Assay Coefficients of Variation for each of the four RT-PCR targets.

(**A**) Intra and Inter Assay Coefficient of Variation (%CV) for snoRNA

| **RT PCR Assay/plate number** | **Ct** | **Average Ct** | **Standard Deviation** | **%CV*** |
| --- | --- | --- | --- | --- |
| **Plate 1** | 26.74 |  |  |  |
|  | 27.33 | 27.03 | 0.41 | 1.53 |
| **Plate 2** | 26.89 |  |  |  |
|  | 27.20 | 27.04 | 0.22 | 0.82 |
| **Plate 3** | 27.04 |  |  |  |
|  | 27.08 | 27.06 | 0.03 | 0.11 |
| **Plate 4** | 26.80 |  |  |  |
|  | 26.80 | 26.80 | 0.00 | 0.01 |
| **Plate 5** | 26.61 |  |  |  |
|  | 26.77 | 26.69 | 0.12 | 0.43 |
| **Inter Assay** |  | 26.93 | 0.23 | 0.84 |

*****%CV calculated on average Ct’s using the equation SD/Average Ct x 100. %CV values were derived from RT-PCR analysis of an RNA sample made up of a pool of controls for all studies.

(**B**) Intra and Inter Assay Coefficient of Variation (%CV) Rn miR 200a

| **RT PCR Assay/plate number** | **Ct** | **Average Ct** | **Standard Deviation** | **%CV** |
| --- | --- | --- | --- | --- |
| **Plate 1** | 27.88 |  |  |  |
|  | 27.90 | 27.89 | 0.01 | 0.05 |
| **Plate 2** | 27.78 |  |  |  |
|  | 27.85 | 27.81 | 0.05 | 0.19 |
| **Plate 3** | 27.76 |  |  |  |
|  | 27.93 | 27.85 | 0.11 | 0.41 |
| **Plate 4** | 27.63 |  |  |  |
|  | 27.53 | 27.58 | 0.07 | 0.25 |
| **Plate 5** | 27.88 |  |  |  |
|  | 27.90 | 27.89 | 0.01 | 0.05 |
| **Inter Assay** |  | 27.78 | 0.14 | 0.50 |

(**C**) Intra and Inter Assay Coefficient of Variation (%CV) Rn miR 200b

| **RT PCR Assay/plate number** | **Ct** | **Average Ct** | **Standard Deviation** | **%CV** |
| --- | --- | --- | --- | --- |
| **Plate 1** | 31.49 |  |  |  |
|  | 31.18 | 31.33 | 0.23 | 0.72 |
| **Plate 2** | 31.51 |  |  |  |
|  | 30.92 | 31.22 | 0.42 | 1.33 |
| **Plate 3** | 30.46 |  |  |  |
|  | 31.38 | 30.92 | 0.65 | 2.11 |
| **Plate 4** | 31.33 |  |  |  |
|  | 31.32 | 31.33 | 0.01 | 0.04 |
| **Inter Assay** |  | 31.20 | 0.35 | 1.13 |

(**D**) Intra and Inter Assay Coefficient of Variation (%CV) Rn miR 429

| **RT PCR Assay/plate number** | **Ct** | **Average Ct** | **Standard Deviation** | **%CV** |
| --- | --- | --- | --- | --- |
| **Plate 1** | 28.42 |  |  |  |
|  | 28.59 | 28.50 | 0.12 | 0.43 |
| **Plate 2** | 28.42 |  |  |  |
|  | 28.56 | 28.49 | 0.10 | 0.36 |
| **Plate 3** | 28.29 |  |  |  |
|  | 28.19 | 28.24 | 0.07 | 0.26 |
| **Plate 4** | 28.23 |  |  |  |
|  | 28.64 | 28.44 | 0.29 | 1.03 |
| **Inter Assay** |  | 28.42 | 0.17 | 0.61 |
